# Improving the application of a practice guideline for the assessment and treatment of suicidal behavior by training the full staff of psychiatric departments via an e-learning supported Train-the-Trainer program: study protocol for a randomized controlled trial

**DOI:** 10.1186/1745-6215-14-9

**Published:** 2013-01-09

**Authors:** Derek P de Beurs, Marieke H de Groot, Jos de Keijser, Bastiaan Verwey, Jan Mokkenstorm, Jos WR Twisk, Erik van Duijn, Albert M van Hemert, Lia Verlinde, Jan Spijker, Bert van Luijn, Jan Vink, Ad JFM Kerkhof

**Affiliations:** 1 Department of Clinical Psychology, VU University, Amsterdam, The Netherlands; 2 EMGO Institute for Health and Care Research, Amsterdam, The Netherlands; 3 GGZ Center for Mental Health Care Friesland, Friesland, The Netherlands; 4 Groningen University, Groningen, The Netherlands; 5 Rijnstate Hospital, Arnhem, The Netherlands; 6 GGZ Center for Mental Health Care GGZ in Geest, 113online, Amsterdam, The Netherlands; 7 GGZ Center for Mental Health Care Delfland, Delft, The Netherlands; 8 Leiden University Medical Center, Leiden, The Netherlands; 9 GGZ Center for Mental Health Care Altrecht, Altrecht, The Netherlands; 10 GGZ Center for Mental Health Care, Propersona, The Netherlands; 11 GGZ Center for Mental Health Care, Dimence, The Netherlands; 12 GGZ Center for Mental Health Care, Rivierduinen, The Netherlands

**Keywords:** Guideline, Implementation, Suicide prevention, Train-the-trainer, E-learning, Healthcare professionals

## Abstract

**Background:**

In 2012, in The Netherlands a multidisciplinary practice guideline for the assessment and treatment of suicidal behavior was issued. The release of guidelines often fails to change professional behavior due to multiple barriers. Structured implementation may improve adherence to guidelines. This article describes the design of a study measuring the effect of an e-learning supported Train-the-Trainer program aiming at the training of the full staff of departments in the application of the guideline. We hypothesize that both professionals and departments will benefit from the program.

**Method:**

In a multicenter cluster randomized controlled trial, 43 psychiatric departments spread over 10 regional mental health institutions throughout The Netherlands will be clustered in pairs with respect to the most prevalent diagnostic category of patients and average duration of treatment. Pair members are randomly allocated to either the experimental or the control condition. In the experimental condition, the full staff of departments, that is, all registered nurses, psychologists, physicians and psychiatrists (n = 532, 21 departments) will be trained in the application of the guideline, in a one-day small interactive group Train-the-Trainer program. The program is supported by a 60-minute e-learning module with video vignettes of suicidal patients and additional instruction. In the control condition (22 departments, 404 professionals), the guideline shall be disseminated in the traditional way: through manuals, books, conferences, internet, reviews and so on. The effectiveness of the program will be assessed at the level of both health care professionals and departments.

**Discussion:**

We aim to demonstrate the effect of training of the full staff of departments with an e-learning supported Train-the-Trainer program in the application of a new clinical guideline. Strengths of the study are the natural setting, the training of full staff, the random allocation to the conditions, the large scale of the study and the willingness of both staff and management to participate in the study.

**Trial registration:**

Dutch trial register: NTR3092

## Background

Suicide is a significant public health issue representing 1.8% of the global burden of disease [1]. The Netherlands ranks among the lower rates with 9.4 suicides per 100,000 inhabitants or approximately 1,500 to 1,600 cases annually [2]. Around 44% of these suicides involve patients in contact with mental health care services [3]. There is limited consensus on how to assess and treat suicidal patients [4]. As a consequence, suicidal patients may not always receive evidence-based care [5,6]. The Dutch Ministry of Health, Welfare and Sports (VWS) commissioned the development of an evidence-based multidisciplinary practice guideline for the assessment and treatment of suicidal behavior (further abbreviated PGSB), which was issued in early 2012 [7]. Adherence to guidelines, however, is not self-evident, due to multiple barriers at both professional and organizational levels. An extensive systematic review of implementation studies identified many barriers at both levels, such as lack of knowledge, and poor outcome expectations at the professional level. Material support, funding and time were found to be common barriers at the organizational level [8].

Grol and Grimshaw found that structured implementation can improve adherence to guidelines [9]. They argue that behavior change is more likely when the intervention is tailored to specific settings and target groups.

A recent systematic review of psychiatric practice guidelines found a modest effect of guideline implementation on provider performance and patient outcome [10]. The authors argue that ongoing support and feedback are effective in changing professional behavior. As suicide prevention in mental health care is essentially the work of multidisciplinary teams [7], and teamwork plays an important role in ensuring patients’ safety and avoiding errors [11], it is advised to train in multidisciplinary teams and to train the full staff of the team [11,12]. When implementing guidelines in a medical setting, a combination of small group interactive postgraduate training, including personalized feedback, and additional instruction material, such as a website, was found to be more successful than a single-faceted intervention [9,13,14]. Finally, e-learning is said to complement face-to-face training in a medical setting; it was found to help medical students become more actively involved in the study material and thereby help to internalize the material [15,16].

Combining these findings, we developed a practical and multifaceted, small interactive group, e-learning supported Train-the-Trainer program (TtT-e) to be delivered to full staff of the departments called PITSTOP suicide (Professionals In Training to STOP suicide). Its content reflects the PGSB. The method of instruction is role play with personalized feedback and instructions by the trainers. The content of the role plays is tailored by the trainers to the specific patient category with which the trainees work on a daily basis in their respective departments. The Train-the-Trainer model of small group interactive educational training is based on Adult Learning Theory [17], which states that people who train others remember 90% of what they teach others, and on the Diffusion of Innovation Theory stating that people adopt new information better through their trusted social networks [18]. The effectiveness of a Train-the-Trainer model is expected since the profits of training by peer-assisted learning in medical health education are comparable to those achieved by professional teachers [19-24]. The Train-the-Trainer program is supported by an e-learning module to complement and prolong the effect of the training.

The PITSTOP suicide study is a cluster randomized controlled trial examining the effect of the TtT-e program on adherence to the PGSB compared with regular guideline dissemination (that is, easy access to guidelines from websites of involved associations, reviews in clinical journals, presentation at conferences, books and manuals), further abbreviated IAU (implementation as usual). The effects are to be examined at the professional and departmental level. We hypothesize that attitudes, knowledge, skills, competence and confidence of mental health care professionals towards suicidal behavior are more likely to improve by the application of the TtT-e program (further abbreviated IAU + TtT-e) than by IAU. Further, we hypothesize that the application of IAU + TtT-e results in better guideline adherence at a departmental level than IAU.

## Method

### Design

This is a multicenter cluster randomized controlled trial in which different psychiatric departments from multiple mental health institutions are clustered and randomized.

### Mental health institution

A mental health institution (MHI) is a regional organization that hosts many different psychiatric departments. In The Netherlands there are 30 MHIs spread throughout the country. Each MHI has its own catchment area of patients and its own professionals [25]. For example, in 2011, MHI Rivierduinen had a catchment area of 1.1 million people within an area of approximately 1,500 square kilometers in the region of South Holland. MHI Rivierduinen hosted 44 different psychiatric departments that together treated 24,753 patients and employed 2,726 employees [26].

### Psychiatric department

A psychiatric department is an independent unit within an MHI. It is an organizational unit with its own management and professional structure. Employees do not work in two departments at the same time. Within one department, patients with various diagnostic categories are treated. However, most departments have one primary diagnostic category that they treat the most, such as depression or personality disorders.

### Recruitment

**Figure 1:**
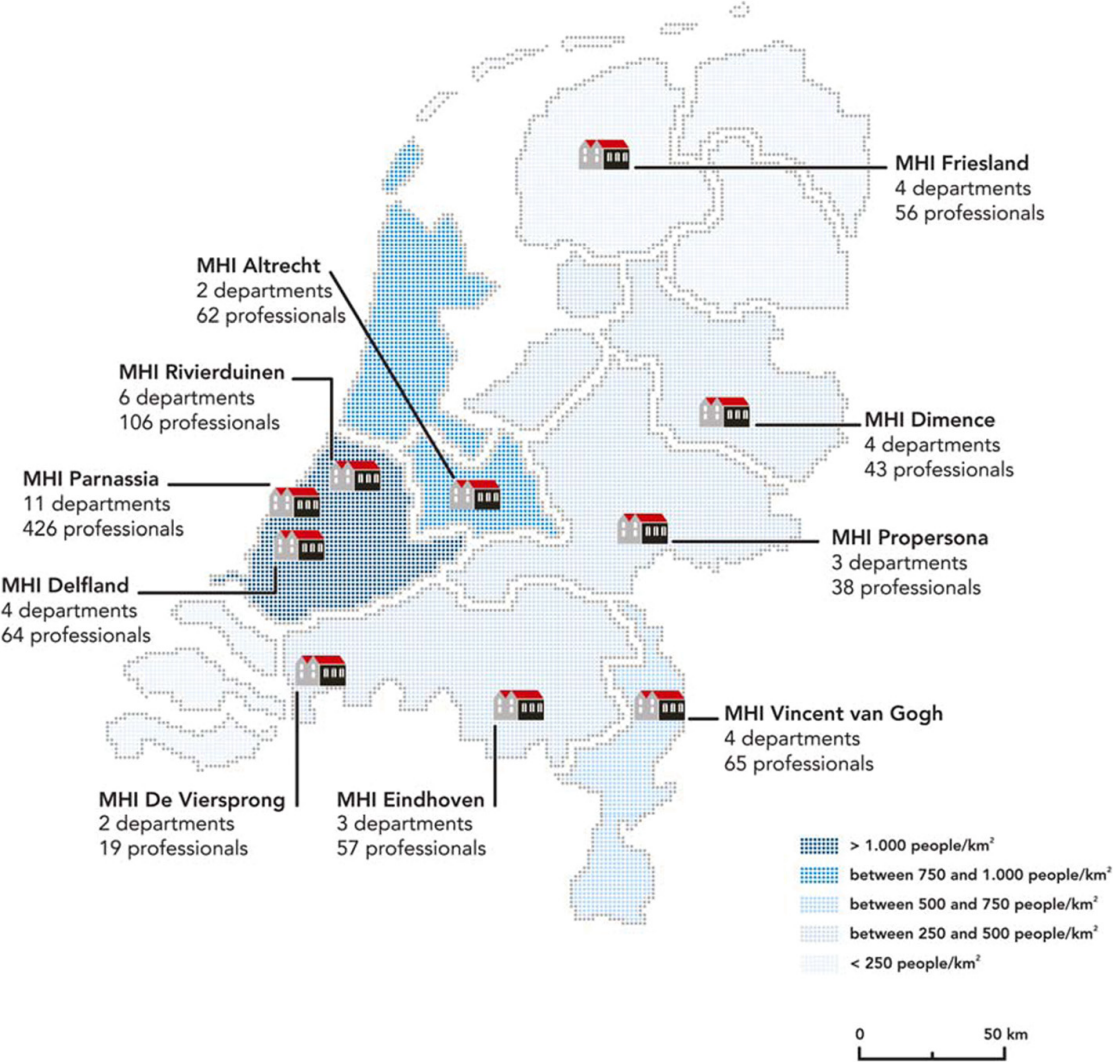
**Overview of the 10 MHIs. **Annotated are the number of departments and the total number of professionals attending PITSTOP suicide per MHI.

Recruitment of MHIs took place during meetings and conferences on suicide prevention in The Netherlands in the two years before the study started. Attending professionals were invited to participate with their MHI in the study. When MHIs expressed willingness to participate, they were requested to indicate at least two departments for inclusion. The MHIs were explicitly asked to provide departments that were hosted in separate buildings or on separate locations with the assurance that staff members were not exchanged or shared in between the departments, to prevent possible exchange of training material. This resulted in 43 participating departments distributed over 10 MHIs (Figure 1).

Departments for adult patient care (>18 years) that were willing to participate were considered eligible for inclusion if: a) the need for training in suicide prevention skills is acknowledged by both the team of professionals and b) by the department’s management, and c) actively supported by the board of the MHI; d) departments are prepared to deal with the demands made by the study (production loss due to the training, requirements for data collection, time needed to study the e-learning module and so on, willingness to be randomized and acceptance of a 50% change of being trained immediately, and a 50% change of a delay of training; e) departments are prepared to meet the demand of 100% participation of individual professionals in multidisciplinary training sessions; f) participating departments were located at separate locations, and that personnel was not exchanged between departments (for example, departments do not share the same psychiatrist).

Each department eligible to join our study was asked to give the following information: prevalence of diagnostic categories, average treatment duration of patients in days, number of new patients admitted a year, and number of registered professionals. For example, an outpatient department of MHI Propersona reported that 70% of their patients have a diagnosis of depression, 10% a diagnosis of borderline, 5% a diagnosis of somatoform disorder, and that 15% have other diagnoses. They treat 200 new patients a year with a team of five psychologists, five psychiatrists and two nurses. The average treatment duration is 365 days. An overview of the information provided by the departments per MHI is shown in Table 1.

**Table 1:** Information received from departments

**Overview departments**						
**MHI Delfland**						
**department**	**profs**	**Nurse**	**Psycho**	**MD**	**average treatment**	**main diagnosis**
**inpatient**	11	8	0	3	1,532	Depression
**outpatient**	24	7	9	7	624	Depression
**Inpatient**	13	11	0	2	1,308	Depression
**Older persons**	16	9	10	8	740	Depression
**MHI Parnassia**						
**department**	**profs**	**N**	**Psy**	**MD**	**average treatment**	**main diagnosis**
**Older persons**	59	36	0	23	21	Depression
**inpatient**	63	50	0	13	16	Depression
**inpatient**	29	18	9	2	250	Personality
**Older persons**	110	80	9	21	41	Depression
**outpatient**	8	5	0	3	90	Depression
**crisis centre**	47	28	0	19	1	Depr/bord/crisis
**inpatient**	36	29	0	7	missing	Depression
**outpatient**	8	0	2	6	240	Addiction
**Older persons**	22	14	3	5	730	Depression
**Older persons**	32	19	5	8	700	Depression
**Older persons**	12	7	3	2	371	Depression
**MHI Eindhoven**						
**department**	**Profs**	**N**	**Psy**	**MD**	**average treatment**	**main diagnosis**
**outpatient**	9	0	4	5	150	Depression
**Inpatient**	32	25	0	7	104	Depression
**outpatient**	16	14	0	2	300	Personality
**MHI Vincent van Gogh**						
**department**	**Profs**	**N**	**Psy**	**MD**	**average treatment**	**main diagnosis**
**outpatient**	13	10	1	2	172	Personality
**neuropsychiatry**	22	14	4	4	96	Mood disorders NOS
**inpatient**	17	14	0	3	20	Schizophrenia
**outpatient**	13	10	0	3	54	Personality
**MHI Dimence**						
**department**	**Profs**	**N**	**Psy**	**MD**	**average treatment**	**main diagnosis**
**inpatient**	16	11	0	4	90	Depression
**outpatient**	13	4	7	2	400	Personality
**outpatient**	9	2	5	2	400	Depression
**outpatient**	5	2	0	3	1	Depr/bord/crisis
**MHI Altrecht**						
**department**	**Profs**	**N**	**Psy**	**MD**	**average treatment**	**main diagnosis**
**Older persons**	20	18	0	2	42	Depr/bord/crisis
**Older persons**	42	26	9	7	150	Depr/bord/crisis
**MHI Friesland**						
**department**	**Profs**	**N**	**Psy**	**MD**	**average treatment**	**main diagnosis**
**Older persons**	24	22	0	2	582	Depression
**Older persons**	18	11	2	5	814	Depression
**Older persons**	7	6	0	1	743	Depression
**Older persons**	7	4	1	2	602	Depression
**MHI Pro Persoona**						
**department**	**Profs**	**N**	**Psy**	**MD**	**average treatment**	**main diagnosis**
**outpatient**	12	9	0	3	1	Depr/bord/crisis
**outpatient**	13	4	0	9	1	Depr/bord/crisis
**outpatient**	13	3	5	5	365	Depression
**MHI Rivierduinen**						
**department**	**Profs**	**N**	**Psy**	**MD**	**average treatment**	**main diagnosis**
**eating disorders**	31	0	23	8	180	Eating disorders
**personality**	20	8	9	3	270	Personality
**inpatient**	15	13	0	2	35	Depression
**personality**	10	5	3	2	240	Personality
**crisis center**	10	Missing	missing	Missing	Missing	Depr/bord/crisis
**Outpatient**	20	Missing	missing	Missing	Missing	Personality
**MHI de Viersprong**						
**department**	**Profs**	**N**	**Psy**	**MD**	**average treatment**	**main diagnosis**
**personality**	14	6	5	3	244	Personality
**personality**	5	1	2	2	240	Personality

*Prof *total number of professionals; *N *number of nurses; *Psy*, number of psychologists; *MD *number of medical doctors; average treatment, average treatment duration of patients in days.

In sum, 936 professionals from 43 participating departments are registered to participate in the study (222 psychiatrists, 130 psychologists, 536 nurses, 48 not defined). Various types of mental health care departments are represented in the study (in- and out-patient care, acute crisis units and long stay departments) treating patients of various diagnostic categories (personality disorder, depressive disorder, anxiety disorder, psychotic disorder).

### Matching procedure

1) The first criterion for matching departments was the most prevalent main diagnostic category. For instance, a department that reported that 60% of their patients had a main diagnosis of depression was matched with another department that reported a comparable percentage of depressive patients. 2) Within groups with comparable diagnostic categories we matched the departments with comparable average treatment duration of patients. We assumed that departments who treat similar types of patients for a similar duration of time are most likely to be comparable on the level of suicidality of their patients. A total of 34 departments were matched according to this procedure. 3) The remaining nine departments differed in a mix of diagnostic categories of patients from other departments so they could not be matched according to the diagnostic criterion. We matched these departments according to comparable treatment duration. We wanted all interested departments that met our inclusion criteria to be able to join the study. By doing so, our study contains a representative sample of all Dutch psychiatric departments that deal with suicidal patients. In the final analysis we will study the sample with and without these last nine departments. We have sent all the pairs to an independent researcher not involved in the study to perform randomization of the matched pairs. Results of the matching procedure and the randomization can be found in Table 2. As our study is performed in a naturalistic setting, we could not match the departments as one can do in a laboratory situation. Still, we believe that our matching procedure is the best possible procedure we could use given the real life conditions. As we matched according to diagnostic criterion and treatment duration, the number of professionals might be uneven as the departments also differed in the number of professionals. A matching procedure where we matched departments on the number of professionals did not work because then the treatment duration and patient categories were unbalanced between pairs. Because of the large number of departments and professionals included in our study we expect that the difference between the number of professionals in the experimental and control conditions will be acceptable. We do not think this will have an influence on the results.

**Table 2:** Results of the matching procedure per department

**Result matching**					
**MHI**	**Department**	**Average treatment**	**Main diagnosis**	**Condition**	**Profs**
**Delfland**	Inpatient	1,532	Depression	EXP	11
**Delfland**	Inpatient	1,308	Depression	CON	13
**Delfland**	Outpatient	624	Depression	EXP	24
**Dimence**	Outpatient	600	Depression	CON	9
**Parnassia**	Outpatient	21	Depression	CON	59
**Parnassia**	Inpatient	16	Depression	EXP	63
**Delfland**	Older persons	740	Depression	CON	16
**Parnassia**	Older persons	730	Depression	EXP	22
**Propersona**	Crisis	1	Depr/bord/crisis	CON	12
**Propersona**	Crisis	1	Depr/bord/crisis	EXP	13
**Parnassia**	Inpatient	250	Personality	EXP	29
**Dimence**	Personality	400	Personality	CON	13
**Dimence**	Crisis	1	Depr/bord/crisis	EXP	5
**Parnassia**	Crisis	1	Depr/bord/crisis	CON	47
**Friesland**	Older persons	582	Depression	EXP	24
**Friesland**	Older persons	814	Depression	CON	18
**Friesland**	Older persons	743	Depression	EXP	7
**Friesland**	Older persons	602	Depression	CON	8
**Parnassia**	Outpatient	90	Depression	EXP	8
**Parnassia**	Outpatient	21	Depression	CON	36
**Viersprong**	Personality	240	Personality	CON	5
**Eindhoven**	Personality	300	Personality	EXP	16
**Eindhoven**	Outpatient	150	Depression	CON	9
**Eindhoven**	Inpatient	104	Depression	EXP	32
**Rivierduinen**	Inpatient	35	Depression	EXP	15
**Rivierduinen**	Outpatient	240	Depression	CON	10
**Parnassia**	Older persons	41	Depression (older persons)	EXP	110
**Parnassia**	Older persons	missing	Depression (older persons)	CON	36
**Dimence**	Inpatient	90	Depression	CON	16
**Propersona**	Outpatient	365	Depression	EXP	13
**Altrecht**	Older persons	42	Depr/bord/crisis	EXP	20
**Altrecht**	Older persons	150	Depr/bord/crisis	CON	42
**Vincent VG**	Outpatient	172	Personality	CON	13
**Vincent VG**	Outpatient	54	Personality	EXP	13
**Rivierduinen**	Personality	270	Personality	CON	20
**Rivierduinen**	Eating disorders	180	Eating disorders	EXP	31
**Viersprong**	Personality	244	Personality	EXP	14
**Parnassia**	Addiction	240	Addiction	CON	8
**Rivierduinen**	Crisis	missing	Depr/bord/crisis	EXP	10
**Rivierduinen**	Personality	missing	Personality	CON	20
**Vincent VG**	Neuropsychiatry	96	Mood disorder NOS	CON	22
**Vincent VG**	Inpatient	20	Schizophrenia	EXP	17
**Parnassia**	Older persons	371	Depression (older persons)	CON	12

Average treatment, average treatment duration of patients in days; *EXP* experimental condition; *CON *control condition; *Profs *total number of professionals.

### Intervention

In the TtT-e program, three types of participants are involved: masters, trainers and trainees. Training is applied on two levels: first, trainers are trained by masters. Subsequently, trainees are trained by trainers. All training sessions are supported by an e-learning module. The TtT-e program as applied by masters is similar to the program applied by trainers.

Masters are experts in the field of suicide prevention due to scientific performance and clinical practice. Trainers are mental health care workers of various disciplines (psychiatrists, psychologists or mental health nurses). They are selected by their management from the clinical staff of departments participating in the PITSTOP-study. Trainers have good training skills, know how to direct role plays, are prepared to train their co-workers and have been selected to serve as a role model on an institutional level and to provide future additional training after all training sessions in the study have been completed. Importantly, they are able to tailor the role plays of the TtT-e program to the needs of the specific patient category of their department. The trainers are instructed to combine the content of the TtT-e program with actual cases from their units. After the study, trainers are expected to continue their role as experts in suicide prevention skills, since on-going support and feedback when implementing psychiatric guidelines are likely to be effective [10].

The TtT-e program reflects the Dutch multidisciplinary guideline on the assessment and treatment of suicidal behavior [7]. The guideline combines the stress-diathesis model [27] and the entrapment model of suicidal behavior [28] to explain the onset and maintenance of suicidal behavior. The combined model depicts suicidal behavior as the outcome of a process that is influenced by the interaction of biological, psychological, environmental and situational factors the interaction of which may lead to entrapment. Entrapment is defined as the specific situation that characterizes suicidal behavior and the specific emotional condition of it. The empirical evidence for the PGSB reflects the international reviews [29-32].

The TtT-e program is supported by two e-learning modules. The first module is developed for the trainees. It consists of video vignettes in which experienced nurses, psychologists and psychiatrists interact with suicidal patients, played by actors, to teach the guideline recommendations. The role playing characters are of various ages, gender and diagnostic categories and they display prototypical suicidal symptoms, cognitions and interaction problems. In between vignettes, guideline topics and recommendations are explained by masters (that is, experts on the topic). Trainees have personalized access to the e-learning module, which can be viewed repeatedly. The total running time of the module is 60 minutes.

In addition to the e-learning module for all the trainees, a second e-learning module was developed specifically for trainers. It provides a video tape of the first training session provided by masters to trainers which was processed into an e-learning format that allows trainers to review the exercises. All trainers were instructed to follow the training protocol manual when training trainees. To survey the adherence to the TtT-e program of trainers, graduate students will randomly visit training sessions. They rate the adherence on a Likert type scale. The scores will be used as a covariate when analyzing the effects of the training.

The difference between an experimental and a control department is that the full staff of the experimental department is trained and the staff of the control department is not trained. The training itself cannot be exchanged between these departments since it consists of a one day training including role playing and direct feedback. What we do not want to be exchanged are the e-learning module, the PowerPoint presentation and written training materials. Professionals in the intervention condition are clearly instructed not to share or distribute training materials with peers, particularly not with peers that work in the control departments. Also, access to the e-learning module is protected with a personalized login code.

### Measurements

**Figure 2:**
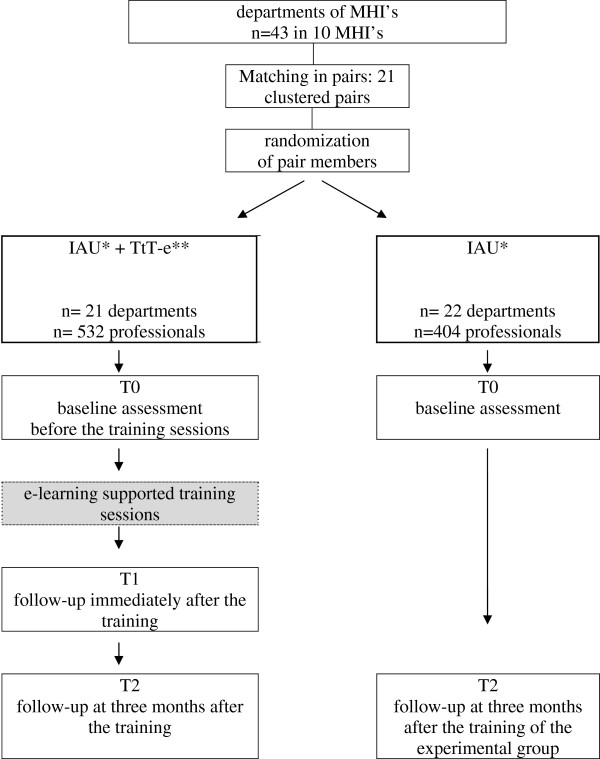
PITSTOP SUICIDE study design. * IAU, implementation as usual. ** TtT-e, e-learning supported Train the trainer program.

The study design and assessment schedule are summarized in Figure 2. The effect of IAU + TtT-e versus IAU will be assessed at the level of professionals and departments.

A link to the assessment will be sent to participants by e-mail. Each participant will be asked to provide an online informed consent form before entering the study.

After completing baseline assessment (T0), participants in the intervention condition have access to the one-day training session and the e-learning module. Follow-up assessment takes place immediately after the training (T1) (participants in the intervention condition) and, subsequently, at three months (T2) after the training. To promote attendance of all professionals in the experimental teams, professional credits, necessary to become or to remain registered as a professional, are awarded.

### Measures at professional level

Measures at professional level concern skills, knowledge, confidence and attitudes of professionals. All scales have been translated into Dutch.

Self-evaluation of knowledge will be assessed by a sub-scale of the 14-item QPR questionnaire (Question, persuade, refer questionnaire [33]). The subscale has been found to measure differences in how professionals rate their knowledge of suicide prevention before and after training [33,34]. Example: “How do you rate your knowledge on suicide prevention?” Scores range from 1(very low) to 5 (very high).

Provider confidence and beliefs is assessed by a six-item questionnaire (Confidence and Beliefs Questions [35]) that has been shown to be able to measure changes in confidence in suicidal behavior management (for example, ‘I am confident in my ability to successfully assess a suicidal patient’). Scores range from 1 (not confident at all) to 5 (very confident).

The ability of professionals to recognize the appropriate response to suicidal patients was measured with the validated 25-item translation [36] of the Suicide Intervention Response Inventory-version 2 (SIRI-2) [37]. Participants have to rate the appropriateness of two “helper” responses on a “client” remark. Rating ranges from −3 (not appropriate at all) to 3 (very appropriate). We use a Visual Analogue Scale instead of the scoring from −3 to 3 to make the SIRI more user-friendly for on-line use.

Guideline adherence by professionals is established by having participants respond to video vignettes in which experienced nurses, psychologists and psychiatrists interact with suicidal characters, played by actors. The video fragments last 30 seconds. Then, professionals are asked to rate the likelihood that they would respond with any of the 25 different interventions on a Visual Analogue Scale (ranging from 1 to 100). For example: ‘Ask whether the patient thinks about suicide’, ‘Ask how hopeless the patient is feeling’. At T0, T1 and T2, the same vignettes are displayed. An expert panel of masters who were involved in the guideline development will also complete the video vignettes. Their scores will serve as reference scores for “excellent guideline adherence”. Scores of participants in the intervention group will be compared with scores of the control group, and with ‘Excellent guideline adherence’ scores of masters. The smaller the difference between the scores by the expert panel and the participant, the better the participant scores on guideline adherence.

Guideline adherence at departmental level will be assessed in both the control and experimental condition one year after the experimental group of the matched pair has completed the PITSTOP intervention. This will be done by NEDKAD (the Dutch Knowledge Center for Anxiety and Depression). For over four years, NEDKAD examines adherence to psychiatric guidelines in different MHIs and departments, using a specific protocol of 10 questions, for example, ‘To what extent is the staff informed about the guideline?’ and ‘How are guideline recommendations translated into practice?’ The NEDKAD procedure is a well-known protocol in The Netherlands for examining guideline adherence in mental health care. It is used as an instrument to assess guideline adherence for the treatment of anxiety and depression. As such, it is used as a regular independent assessment of the quality of care of departments specialized in the treatment of anxiety and depression. It is accepted by the Dutch MHI centers. Ten questions are being answered and scored by two independent assessors. The approach has not yet been published. We aim to further develop and systematize the instrument for measuring adherence to the suicide guideline. We will collaborate with NEDKAD.

### Statistical analysis

Our study is a design with different levels. For the outcomes on the professional level we have repeated observations (level 1) that are nested within trainees (level 2). Trainees are nested within trainers (level 3), trainers within departments (level 4) and departments are nested within MHIs (level 5). For the analysis on an organizational level we have departments that are nested within MHIs. Multilevel models are hierarchal systems that estimate random coefficients and variance components for each level. Random intercepts will be included in the multilevel models [38].

Approval from the Medical Ethics Committee of the VU University Medical Center (registration number 2011/151) was requested and obtained. We have sent the advice from the Medical Ethics Committee to the ethical committees of the participating departments so they could give their own reaction. All other committees agreed with the Ethics Committee of the VU.

## Discussion

This paper describes the study protocol of a cluster randomized controlled trial measuring the effects of a practical and multifaceted, e-learning supported Train-the-Trainer model at the professional and departmental level. Strengths of the study are the natural setting, the training of the complete staff in the experimental condition, the random allocation to dissemination conditions, the large scale of the study, and the willingness of both staff and management to participate in the study.

Spill-over of training material to the control conditions is a potential risk of our design. By making sure that experimental and control departments are on different locations and do not share personnel, by restricting the e-learning module with a personal login code and by instructing trainees not to spread any material among peers in the control condition, we minimize contamination.

Up until now, willingness of both management and employees to participate in the TtT-e program is excellent. Boards of the participating centers provided consent and reserved time to elaborate upon the project with the research team. The first training was given in January 2012, and the final training will be given around November 2012. Results will be available in the beginning of 2013.

We included many different departments in our study. What all departments have in common is that they have a bottom-up wish for improving their skills in suicide prevention. From the literature we know that this is an important factor for success [12]. As a result of this study, we will know that for groups with a bottom-up need for training and top-down support, PITSTOP suicide is or is not an effective method to implement the guideline among professionals.

## Abbreviations

NEDKAD: The Dutch Knowledge Center for Anxiety and Depression; IAU: Implementation as usual; MHI: Mental Health Institution; PGSB: Multidisciplinary practice guideline for the assessment and treatment of suicidal behavior; PITSTOP: Professionals In Training to STOP suicide; TtT-e: E-learning supported Train-the-Trainer program; VWS: Dutch Ministry of Health Welfare and Sports.

## Competing interests

The authors declare that they have no competing interest.

## Authors’ contributions

AK, MdG, JM and JdK obtained funding for this study. DP and MdG drafted the manuscript and will carry out the study. DP designed the e-learning modules. MdG and AK designed the TtT-e protocol; AK, BH and BV contributed to writing the manuscript. All authors contributed to the execution of the study, and approved the final draft.
